# Corneal in vivo confocal microscopy to detect belantamab mafodotin-induced ocular toxicity early and adjust the dose accordingly: a case report

**DOI:** 10.1186/s13045-021-01172-5

**Published:** 2021-10-03

**Authors:** Kevin Marquant, Anne Quinquenel, Carl Arndt, Alexandre Denoyer

**Affiliations:** 1 University Hospital of Reims, Reims, France; 2 University of Reims Champagne-Ardenne, Reims, France; 3 Institut de la Vision, U968, Sorbonne University, Paris, France; 4 Research Team CARDIOVIR, EA4684, University of Reims Champagne-Ardenne, Reims, France; 5 Robert Debré University Hospital, Rue du General Koenig, 51100 Reims, France

**Keywords:** Multiple myeloma, Antibody–drug conjugate, Ocular toxicity, Microcystic keratopathy, In vivo confocal microscopy

## Abstract

**Background:**

New targeted antibody–drug conjugates (ADCs) against multiple myeloma are known to induce adverse effects that may lead to treatment discontinuation. Preclinical studies reported early severe ocular damage related to the use of belantamab mafodotin (belamaf), including ocular surface inflammation, severe dry eye, and a specific toxicity to the cornea, namely microcystic keratopathy. While belamaf-induced ocular changes have not been prospectively studied, a better understanding of mechanisms involved as well as kinetics may aid in anticipating dose adjustment rather than stopping the treatment once clinical ocular damage is too severe.

**Case presentation:**

A 61-year-old woman scheduled for belamaf as a fifth-line treatment against multiple myeloma was prospectively included. Clinical examinations were performed before and every 3 weeks afterward, together with in vivo confocal microscopy (IVCM) of the cornea. Visual acuity, symptoms, slit-lamp examination, and ultrastructural changes of the cornea were recorded according to the received dose of belamaf. More precisely, kinetics, shape, density, and location of the toxic corneal lesions have been followed and analyzed using IVCM. Also, specific lesions at the sub-basal nerve plexus layer were detected and characterized for the first time. This advanced approach allowed a better understanding of the belamaf-induced toxicity, further balancing the dose to maintain good vision and eye health while continuing the treatment.

**Conclusions:**

Systematic ultrastructural analysis and follow-up of the corneal state during ADCs treatment for multiple myeloma may open new avenues in the therapeutic approach. Early preclinical detection of ocular damage may accurately contribute to finding the correct dose for each patient and not stopping the treatment due to severe ocular adverse effects.

**Supplementary Information:**

The online version contains supplementary material available at 10.1186/s13045-021-01172-5.

## Background

Novel targeted immunotherapies currently developed against multiple myeloma (MM) may provide a more profound and durable response than conventional treatments [1]. Belantamab mafodotin (belamaf; GSK2857916, GSK, Brentford, UK) is an innovative targeted antibody–drug conjugate (ADC) made of a humanized IgG1 monoclonal antibody against the B-cell maturation antigen conjugated with a tubulin polymerization inhibitor agent (Monomethyl Auristatin-F [MMAF]) [2]. Preclinical and clinical studies demonstrated promising results in patients with relapsed/refractory MM [3–6]. However, serious adverse events have been reported early, including vision disturbance, dry eye disease, and corneal disease [7–10], with an incidence of corneal damage up to 70% [5]. Few things are known about the space–time kinetics of the corneal ultrastructural changes induced by belamaf. The particular belamaf-induced corneal disease, microcystic keratopathy, is a new entity that is highly specific to ADCs conjugated to MMAF. It has been retrospectively studied in the phase II study patients, further emphasizing the need for additional research [11]. Indeed, a better understanding of this specific corneal toxicity and close collaboration between ophthalmologists and hematologists are crucial for conducting optimal treatment. We herein report an ultrastructural follow-up of the corneal damage in a patient treated with belamaf that reveals specific lesions in relation to the patient’s symptoms and may contribute to an improved way to anticipate dose adjustment according to in vivo confocal microscopy (IVCM) corneal imaging.

## Case presentation

A 61-year-old Caucasian woman receiving belamaf as a fifth-line treatment for refractory MM was prospectively included. Institutional Review Board approved the follow-up, and the patient gave informed consent.

The patient presented no relevant medical history. Ophthalmological examinations were performed before and every 3 weeks during 15 weeks of treatment, then 6 months after treatment discontinuation due to therapeutic failure. Symptoms (Common Terminology Criteria for Adverse Events, CTCAE), visual acuity, and clinical signs have been collected. In parallel, IVCM of the cornea was performed at each visit for ultrastructural imaging. Briefly, the superficial epithelium (5 µm deep), the basal epithelium (40 µm deep), and the sub-basal nerve plexus layer (anterior stroma, 55 µm deep at the level of nerve plexus under the Bowman’s layer) were scanned in the periphery (4 images per layer, i.e., one per quadrant at 1 mm from the limbus) and in the center (4 images per layer) of the cornea. Sectional view of the anterior layers of the cornea is presented in Additional file 1: Fig. S1. Axial and frontal locations, density, size, and circularity of the belamaf-induced corneal lesions were analyzed and averaged using ImageJ software (NIH, USA). All these data are detailed in Table 1, together with the dose of belamaf.

**Table 1:** Symptoms, clinical signs, and in vivo confocal microscopy of the cornea in the worst eye of a patient treated with belantamab mafodotin

Time (weeks)	Pre-	3	6	9	12	15	36
*Symptoms, vision, and slit-lamp examination of the cornea*							
Blurred vision (CTCAE, 0–4)	0	0	3*	0	1	1	0
Photophobia (CTCAE, 0–4)	0	1	2	0	0	0	0
Visual acuity	20/20	20/20	20/40*	20/20	20/25	20/25	20/20
SPK (0–5)	0	1	3*	2	2	2	0
Microcysts	None	None	Diffuse	Diffuse	Diffuse	Diffuse	None
In vivo* confocal microscopy of the cornea. Location: peripheral/central*							
Epithelial microcysts							
Density (/mm^2^)			25/6.3	6.3/12.5	0/6.3	62.5/62.5	0/0
Average size (μm)	0/0	0/0	10.7/14.3	18.4/14.7	0/14.7	18.8/15.7	
Circularity (0–1)			0.67/0.9	0.88/0.94	/0.94	0.77/0.84	
Hyperreflective deposits: superficial epithelium							
Density (/μm^2^)		31.3^§^/0	138/87.5	62.5/81.3	56.3/62.3	62.5/106	
Average size (μm)	0/0	8.5/na	6.8/7.5	7.1/9.1	7.6/7.4	8.8/9	0/0
Circularity (0–1)		0.72/na	0.67/0.69	0.72/0.80	0.62/0.65	0.81/0.83	
Hyperreflective deposits: basal epithelium							
Density (/μm^2^)		68.8^§^/0	381/219	343/119	131/93.8	93.8/206	
Average size (μm)	0/0	9.0/na	9.9/8.9	6.9/9.1	8.6/7.2	8.7/9.2	0/0
Circularity (0–1)		0.56/na	0.61/0.64	0.75/0.75	0.63/0.59	0.67/0.74	
Hyperreflective deposits: sub-Bowman nerve plexus layer (anterior stroma)							
Density (/μm^2^)		100^§^/0	225/169	62.5/50	37.5/68.8	75/31.3	
Average size (μm)	0/0	6.4/na	8.2/8.9	8/6.3	7.5/6.4	6.6/7.9	0/0
Circularity (0–1)		0.66/na	0.61/0.64	0.48/0.64	0.64/0.72	0.69/0.59	
*Treatment (belantamab mafodotin)*							
Delivered dose after exams (mg/kg)	2.5	2.5	0	1.9	1.9	0	0
Cumulative dose (mg)	0	180	360	360	490	620	620

*CTCAE* common terminology criteria for adverse events, *na* not applicable, *SPK* superficial punctuate keratitis. (Oxford’s score)

^*^Clinical ocular features that influenced the dose adjustment/temporary discontinuation of belantamab mafodotin

^§^IVCM putative preclinical markers for ocular toxicity (before any clinical signs/symptoms)

**Fig. 1:**
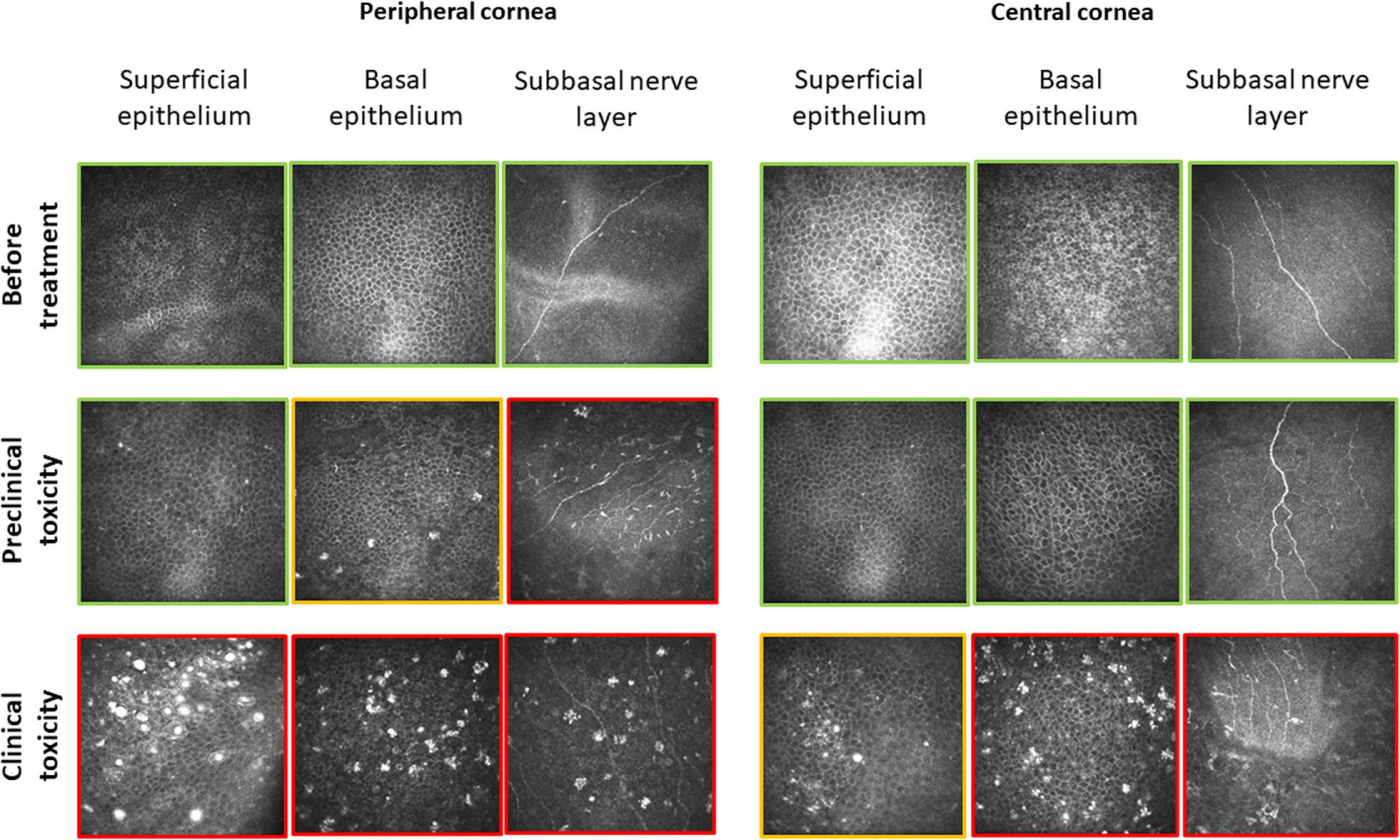
In vivo confocal microscopy of the peripheral and central cornea at various depths, according to belamaf ocular toxicity stage. Before being able to note any clinical signs or vision loss, a high density of hyperreflective deposits is detected specifically in the deeper layers of the peripheral cornea (red square for high density in the sub-Bowman’s layer and an orange square for low density in the basal epithelium). At more severe stages (clinical signs, vision loss), hyperreflective deposits are detected in every layer and peripheral and central areas. Deep deposits appear as a “bunch of grapes” shaped lesions, which transform into round intraepithelial microcysts the closer they go to the surface

At the pre-treatment visit, no corneal clinical findings nor IVCM abnormalities were found (Fig. 1, first line). Mild-severity dry eye disease was diagnosed and treated with tear film substitutes. Hematologists were informed of the ocular state, and the first dose of belamaf (2.5 mg/kg, 180 mg) was administered.

At the week three visit, she reported no symptoms but mild photophobia, without any consequences on visual acuity. Slit-lamp examination found only low-severity punctuate keratitis in the peripheral cornea. In contrast to the vision and examination, IVCM revealed the emergence of significant clusters of hyperreflective material mainly localized around the Bowman’s layer, i.e., at the basal epithelium and the sub-basal nerve plexus layer, which were localized in the peripheral cornea (Fig. 1, second line). Superficial epithelium at the periphery as well as any layer of the central cornea was free of changes as assessed by IVCM. A second full dose treatment was then administered.

**Fig. 2:**
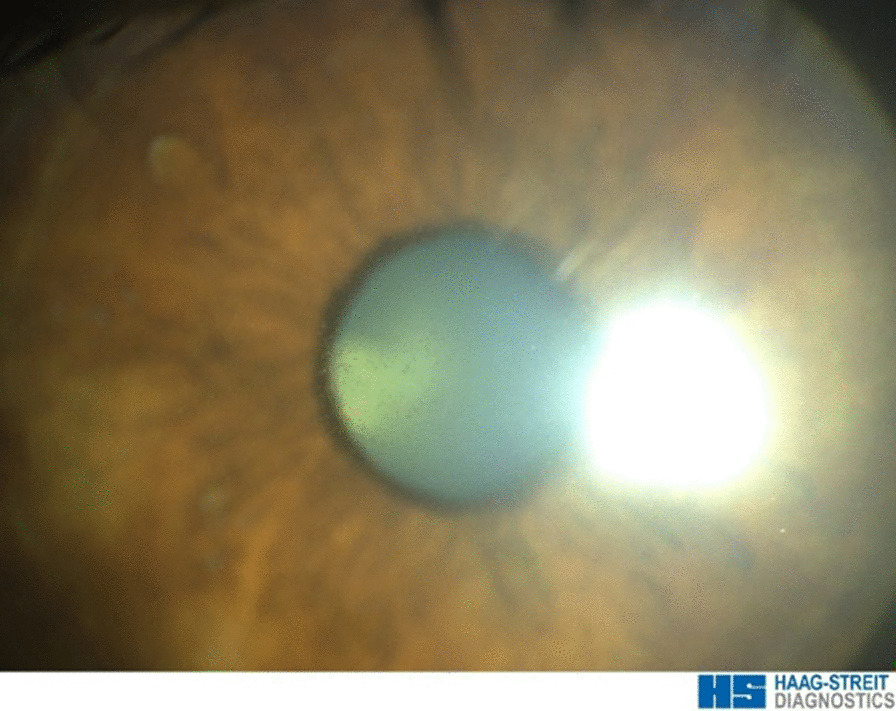
Diffuse microcystic keratopathy as observed by slit-lamp examination at week six. Belamaf induces a toxic corneal disease, microcystic keratopathy, specific to ADCs with monomethyl auristatin-F

At week six, symptoms dramatically increased, including blurred vision and decreased best-corrected visual acuity of 20/40 (loss of 5 lines) in the left eye and 20/25 (loss of 3 lines) in the right one. Slit-lamp examination revealed an increase in superficial punctuate keratopathy, as well as a microcystic keratopathy from the limbus to the center of the cornea (Fig. 2). IVCM imaging found a worsening of the corneal changes with a higher density of hyperreflective deposits in the basal epithelium and the sub-basal nerve plexus layer, diffusely over the entire corneal surface forming real “bunch-of-grapes” shaped clusters. These were also found at the superficial layer of the epithelium for the first time (Fig. 1, third line). The changes took on a balloon appearance at a more superficial level, forming small degenerative intraepithelial microcysts, mainly consisting of a hyper-reflective wall. The latter was present in large quantities at the corneal periphery level and, to a lesser extent, at the cornea center. Based on the vision loss, clinical keratitis, and central lesions assessed by IVCM, it was decided to stop the treatment.

**Fig. 3:**
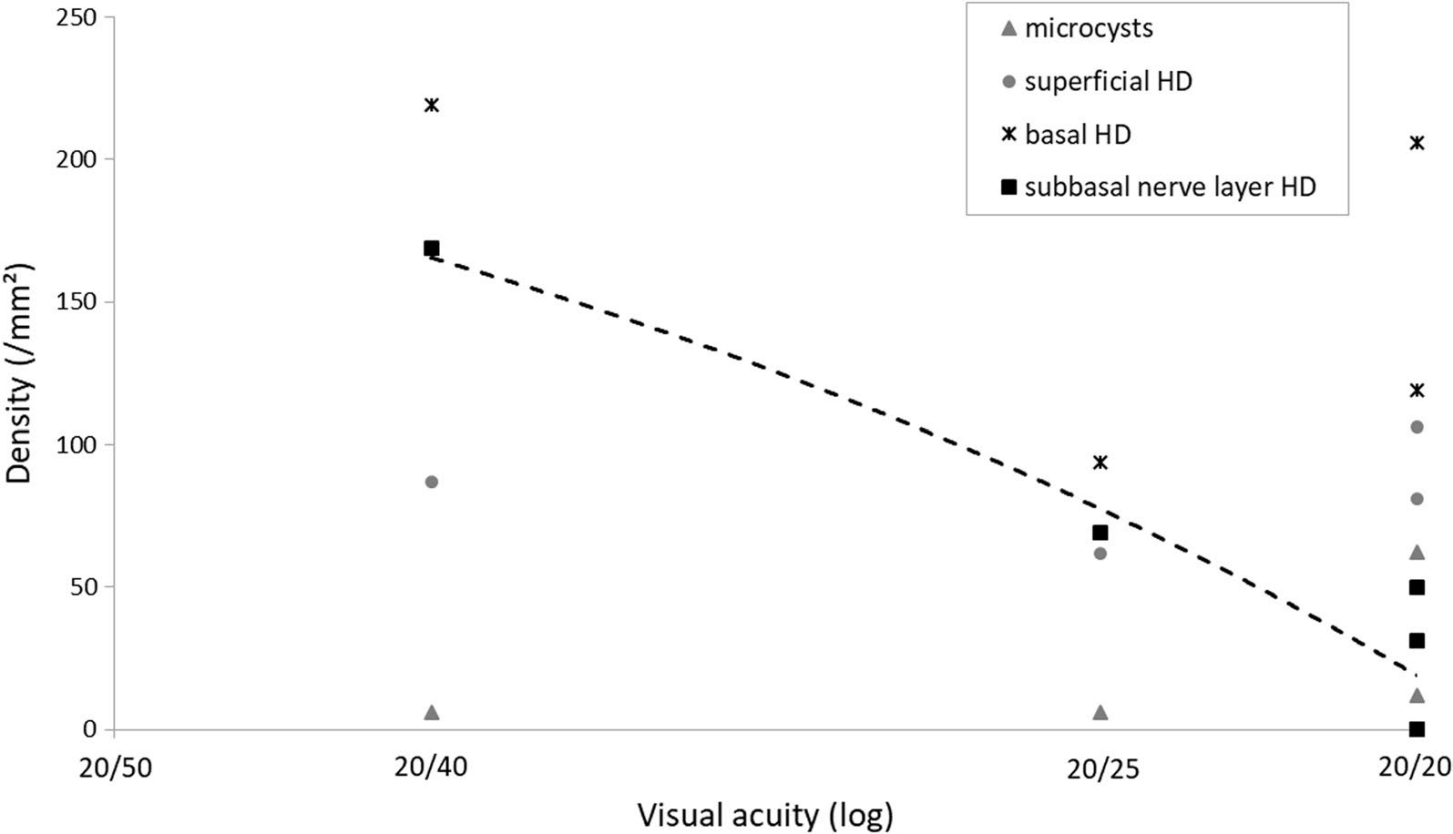
The density of belamaf-induced corneal lesions (assessed by in vivo confocal microscopy) as a function of the visual acuity. The density of hyperreflective deposits (HDs) in the sub-basal nerve plexus layer seems to correlate with vision loss. In contrast, HDs in the basal and superficial epithelium and superficial microcysts do not

Three weeks after treatment discontinuation, the patient no longer reported a functional complaint. Visual acuity had returned almost to baseline. However, the microcystic keratopathy seemed to be clinically more crucial, in contradiction to the recovery of visual acuity and symptoms. This intriguing fact was explained by corneal IVCM, partially at least, which showed a total wash-out of the hyperreflective lesions in the deeper cornea (basal epithelium and sub-Bowman layer). In contrast, microcysts and deposits were still at the surface. Indeed, vision loss seemed to depend mainly on the density of the “bunch-of-grapes” shaped deposits at the sub-basal level, as illustrated by Fig. 3. Based on the deep clearance of the corneal lesions assessed by IVCM, it was decided to resume the treatment with belamaf at 75% of the dose (1.9 mg/kg, 130 mg).

**Fig. 4:**
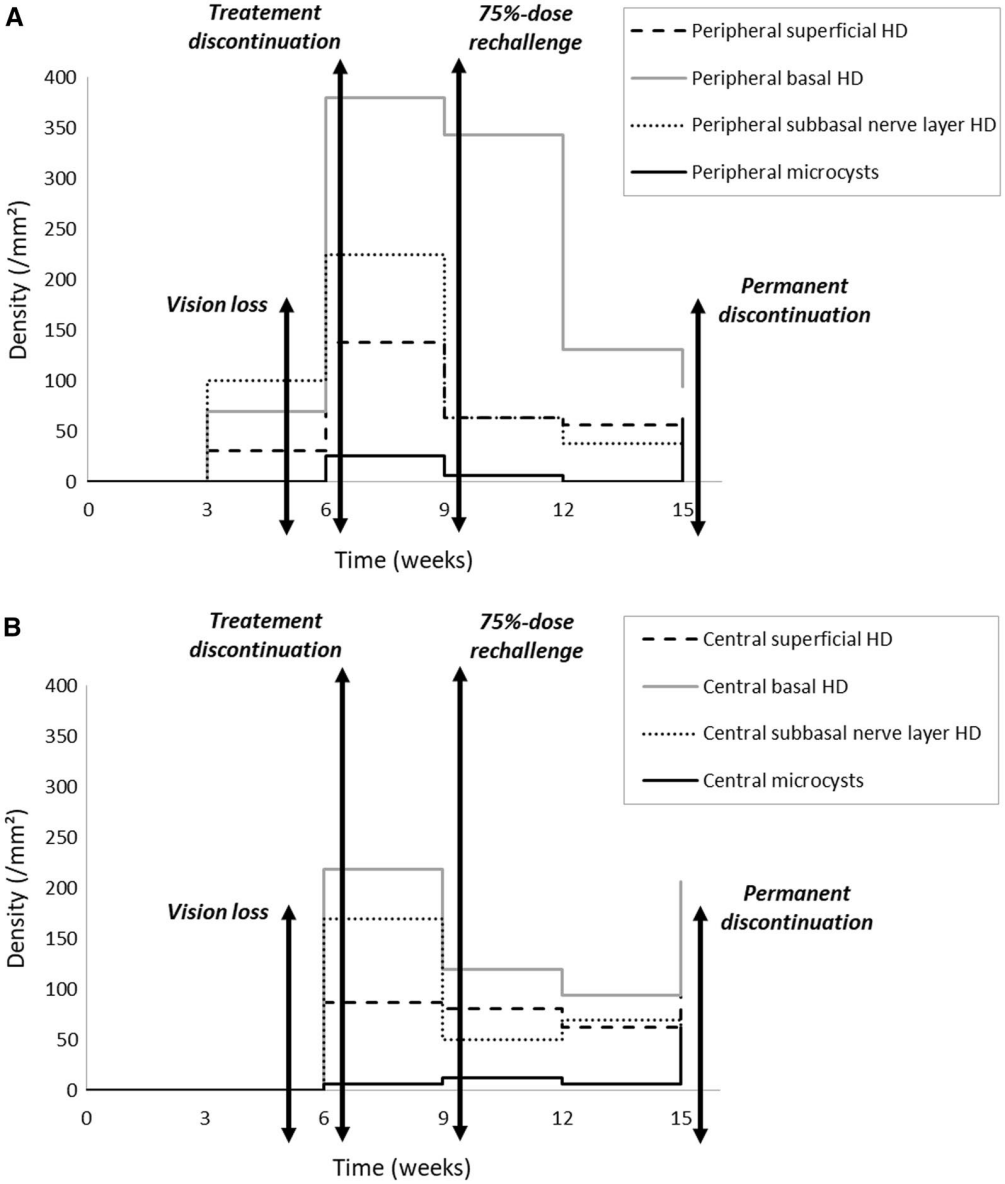
Follow-up of belamaf-induced ocular toxicity (assessed by in vivo confocal microscopy of the cornea) in relation to symptoms and treatment management. **a** Peripheral cornea: specific hyperreflective deposits (HDs) are detected in basal layers at W3 before vision loss; these may constitute preclinical biomarkers for ocular toxicity. **b** Central cornea: belamaf-induced lesions are detected simultaneously as the vision loss and clinical signs; rechallenging with 75% of the dose contributed to decreasing the density, further keeping corneal toxicity under control. Permanent discontinuation was driven by general treatment failure

Symptoms, vision, and clinical findings were primarily stable at week nine and week 12. IVCM revealed (1) an overall decrease in microcysts and hyperreflective deposits density in the entire cornea, but (2) new deposits at the basal epithelium and the sub-Bowman layer, which progressively went from the periphery toward the center (Fig. 4). Their density, however, was lower than what was previously observed with 100% of the belamaf dose, even if some superficial microcysts appeared as “balloon release” at week 12. Unfortunately, at the end of this fifth cycle (week 15), the hematology department decided to stop the treatment due to MM progression.

A complete ophthalmological examination was carried out 6 months after the treatment discontinuation. The patient presented no symptoms, the visual acuity had returned to baseline, and the clinical examination was normal. The entire corneal tissue was sound, as assessed by IVCM.

## Discussion

For the first time, prospective monitoring of the cornea by IVCM allowed a better understanding of the nature, kinetics, and dose-dependency of belamaf-induced ocular toxicity at the microscopic level, together with its consequences on vision and clinical signs. The ocular toxicity of belamaf is already known. However, belamaf-induced or other ADC-induced corneal lesions are often not accurately studied in clinical trials, and the methods are vague. Indeed, very few studies aimed at systematically evaluating and following the corneal toxicity with specific imaging devices of the cornea; it is summarized in Table 2.

**Table 2:** Previous studies using corneal imaging to describe belamaf-induced and other antibody–drug conjugate-induced ocular toxicity

ADC/disease	Authors	Design (n)	Imaging	Findings and limitations
Belantamab mafodotin/MM	Farooq et al. [11]	Retrospective review in the patients’ cohort DREAMM-2. (72)	IVCM	Epithelial microcysts and basal opacities
				Largest series, but few IVCM examples
				No quantification
				No space–time kinetics
Belantamab mafodotin/MM	Matsumyia et al. [17]	Case report (2)	OCT	Hyperreflective lesions in some epithelial areas
				Low-resolution imaging
Belantamab mafodotin/MM	Rousseau et al. [10]	Image (1)	IVCM	Epithelial microcysts
				No quantification
				No space–time kinetics
EGFR-inhibitor ABT-414/glioblastoma	Parrozzani et al. [14]	Prospective analysis (10)	IVCM	Epithelial microcysts and subbasal nerve plexus disappearance
				Well detailed but no quantification
				Space–time kinetics done
Trastuzumab emtansine/breast cancer	Deklerck et al. [15]	Cross-sectional prospective (12)	IVCM	Microcysts mainly in the peripheral epithelium
				No quantification
				Temporal stability of the lesions
Trastuzumab emtansine/breast cancer	Kreps et al. [16]	Case report (1)	IVCM	No initial images
				Epithelial microcysts and basal deposits
				No quantification
				Stability of the lesions
Mirvetuximab soravtansine/ovarian, peritoneal, and fallopian tube cancer	Corbelli et al. [18]	Case report (5)	OCT and AS-IR	Subepithelial changes and corneal hyporeflective dots
				Low-resolution imaging
				No spatial quantification
				Space–time kinetics done

*ADC* antibody–drug conjugate, *AS-IR* anterior segment-infrared reflectance, *IVCM* in vivo confocal microscopy, *MM* multiple myeloma, *OCT* optical coherence tomography

The first key point of our findings is a specific tropism of belamaf toxicity at the basal epithelium and under Bowman’s layer at the sub-basal nerve plexus level. In this specific place, belamaf-induced changes appeared originally as hyperreflective “bunch-of-grapes” shaped deposits, which became the most crucial factor for vision loss (Fig. 3). The evolution of corneal changes over time also revealed a migration of deposits to the outermost epithelial layers and from the periphery to the center of the cornea, together with fairly rapid transformation into round intracellular microcysts. Clinical microcystic keratopathy is reportedly a specific side effect of ADCs using MMAF or a related molecule [9, 12, 13]. Visual acuity and clinical microcystic keratopathy are the two endpoints taken into account during follow-ups in the dosage management recommendations for belamaf [11]. The present case shows that impaired vision is mainly related to an accumulation of deep toxic deposits in the central epithelium as assessed by IVCM, more so than microcystic keratopathy.

The exact mechanism behind the corneal side-effect of belamaf is still unknown, but several hypotheses have been proposed. Many ADCs (such as trastuzumab emtansine, ABT-414, or mirvetuximab soravtansine) present corneal toxicity also [14–18]. It raises the question of the respective involvement of the payload and the target. Specific toxicity of the payload may be involved in ocular toxicity of belamaf since other MMAF-containing ADCs induce corneal damage in about the same proportion, further sharing some common ultrastructural characteristics [3, 4, 9]. However, other ADCs containing emtansine or sorvastatine also present corneal toxicity. On-target mechanism, as it is the case for trastuzumab emtansine [15, 16], and off-target mechanism are involved in the pathogenesis of ADC toxicity. For belamaf, one hypothesis would be direct toxicity by binding to a basal epithelial corneal cell antigen (BCMA), which is said to be similar to the ADC’s target antigen [14]. BCMA is not an antigen known to be present in the basal cells of the corneal epithelium. However, we could assume the existence of another phenotypically similar surface protein on which belamaf could bind and exert direct toxicity. The hypothesis of an off-target mechanism is much more relevant, since belamaf and the other MMAF-containing ADCs may enter the cornea via micropinocytosis [12]. Non-specific absorption of the ADC within the proliferating limbic stem cells would lead to mitotic process alterations and further causing cell degeneration [8, 9, 14]. Normal renewal of corneal epithelium would allow the lesions to regress after stopping treatment [19]. This would explain the spatial kinetics of the lesions observed in our study using IVCM, an evolution from the periphery to the center and from the basal layers to the superficial ones, reflecting basic turnover/renewal of the corneal epithelium. Indeed, Bausell et al. recently detailed such a turn-over of the belamaf-induced corneal lesions by conducting a clinical follow-up [20]. Last, passive diffusion of the active molecule once internalized within basal epithelium layers could be discussed [9].

We also found that the sub-basal nerve plexus layer—in the anterior stroma just below the basal epithelium—was specifically involved during belamaf ocular toxicity. Parrozzani et al. showed corneal nerve fragmentation and keratocyte activation in some patients treated with another ADC [14]. Our study did not observe significant changes in the density or morphology of the nerve plexus, nor did we observe keratocyte activation in the corneal stroma as assessed by IVCM. However, it must be noted that the treatment had been initially stopped for three weeks then resumed at 75% of the dose, which may have prevented more extended toxicity, such as neuronal or stromal damage. These points should be carefully studied in the future.

The second important finding of this case relies on the ability of IVCM to diagnose belamaf toxicity at a preclinical stage (Fig. 4a). Indeed, deep hyperreflective deposits were observed in the peripheral cornea 3 weeks after the first 100%-dose injection, whereas poor clinical signs and no vision loss had occurred. Corneal damage results probably from purely toxic mechanisms, and the use of anti-inflammatory eye drops has been proven to be ineffective for prophylactic and therapeutic purposes [5, 21]. It should be noticed that we did not observe any immune cell infiltration nor dendritic cells whatever the corneal layer, further highlighting the lack of inflammatory processes. Temporarily discontinuing the therapy is the only way recommended to manage clinically severe ocular toxicity. In the present case, a decrease in visual acuity associated with severe keratitis led to temporary discontinuation of treatment with belamaf, according to the currently recommended management techniques [11]. Simultaneously, IVCM revealed a massive density of hyperreflective deposits, especially around the Bowman’s layer in the central cornea, which preexisted, interestingly, 3 weeks earlier in the peripheral cornea. Following the natural history and turnover of the belamaf-induced corneal lesions, we could hypothesize that a certain level of peripheral deep deposits may trigger subsequent severe corneal damage that leads to treatment discontinuation. As a result, the deep peripheral density of belamaf-induced hyperreflective deposits assessed by IVCM could be a putative marker for tapering off the treatment before major ocular issues occur to prevent discontinuation. Accordingly, in this case, treating again with 75% of the theoretical dose seemed to control the corneal toxicity and did not lead to severe corneal damage nor deep vision loss, further reflecting dose-dependent toxicity.

## Conclusions

The present case reinforces that close collaboration between hematologists and ophthalmologists would clearly benefit patients treated with ADCs. Monitoring the cornea by IVCM, in the case of MM treated with belamaf, demonstrates that IVCM may (1) aid to better understand the ocular toxicity induced by belamaf, and (2) contribute to the management of improved treatment and adjustment of the dose according to a targeted ultrastructural follow-up of the deep peripheral cornea. IVCM enabled the characterization of the lesions and their corneal turnover throughout the treatment. We suggest that specific lesions at the sub-basal nerve plexus layer can be used as preclinical biomarkers of toxicity to conduct the best treatment by anticipating dangerous ocular side-effects. Prospective clinical studies with a frequent and regular follow-up of belamaf-induced corneal changes using IVCM should contribute to defining the key role of this device in the recommended management of patients treated with belamaf or other ADCs.

## Supplementary Information

**Additional file 1: Figure S1** Side-cut of the anterior layers of the cornea and the corresponding “en face” images using in vivo confocal microscopy.

## Data Availability

The datasets are available from the corresponding author (Prof. Alexandre DENOYER, alexandre.denoyer@gmail.com) on reasonable request.

## Abbreviations

ADC: Antibody–drug conjugate
IVCM: In vivo confocal microscopy
MM: Multiple myeloma
MMAF: Monomethyl auristatin-F
