# Supplementary figures and images for: Corneal in vivo confocal microscopy to detect belantamab mafodotin-induced ocular toxicity early and adjust the dose accordingly: a case report
Source: J Hematol Oncol. 2021 Oct 3;14:159. doi: 10.1186/s13045-021-01172-5 (PMC8489063; doi:10.1186/s13045-021-01172-5)

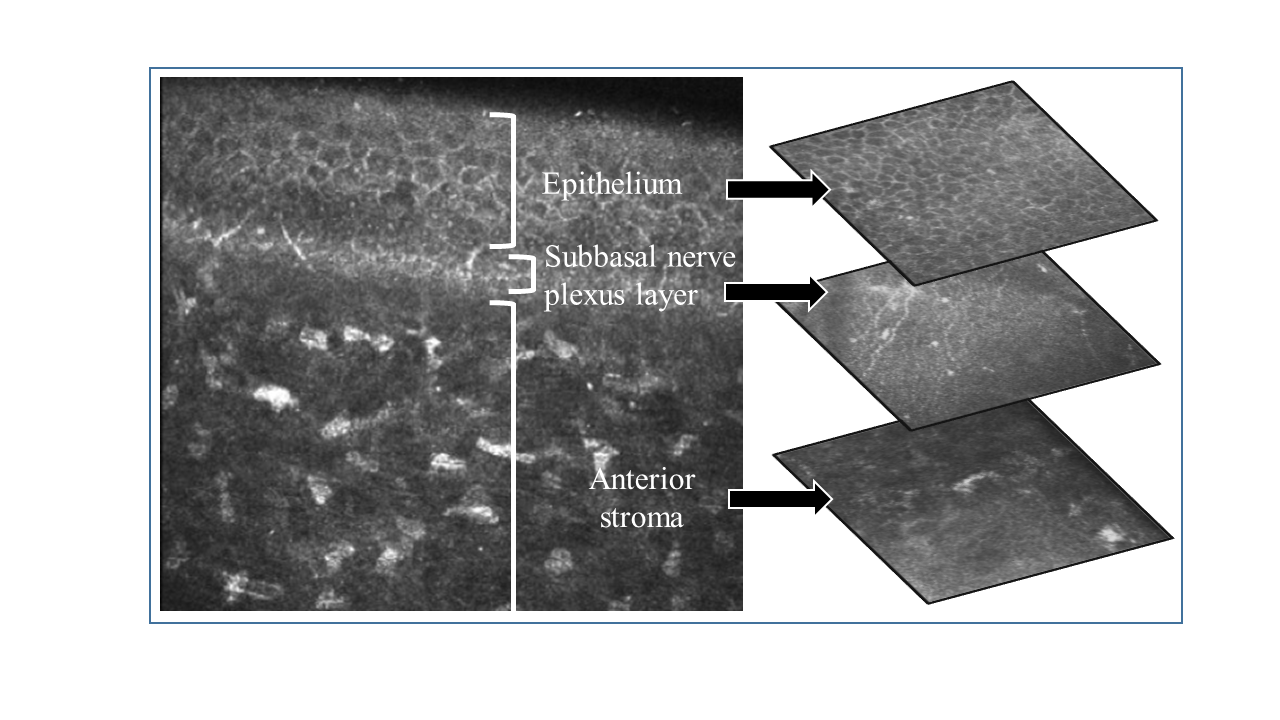

Supplement: Supplementary file 1 — Additional file 1: Figure S1 Side-cut of the anterior layers of the cornea and the corresponding “en face” images using in vivo confocal microscopy. [file 13045_2021_1172_MOESM1_ESM.tif]
